# Reliable reference genes for expression analysis of proliferating and adipogenically differentiating human adipose stromal cells

**DOI:** 10.1186/s11658-019-0140-6

**Published:** 2019-02-15

**Authors:** Claudia Krautgasser, Markus Mandl, Florian M. Hatzmann, Petra Waldegger, Monika Mattesich, Werner Zwerschke

**Affiliations:** 1 Division of Cell Metabolism and Differentiation Research, Institute for Biomedical Aging Research, University of Innsbruck, Rennweg 10, A-6020 Innsbruck, Austria; 2 Department of Plastic and Reconstructive Surgery, Innsbruck Medical University, Anichstraße 35, A-6020 Innsbruck, Austria

**Keywords:** Adipose stromal cells, Adipose stem cells, Adipogenesis, Proliferation, Differentiation, Reference gene

## Abstract

**Background:**

The proliferation and adipogenic differentiation of adipose stromal cells (ASCs) are complex processes comprising major phenotypical alterations driven by up- and downregulation of hundreds of genes. Quantitative RT-PCR can be employed to measure relative changes in the expression of a gene of interest. This approach requires constitutively expressed reference genes for normalization to counteract inter-sample variations due to differences in RNA quality and quantity. Thus, a careful validation of quantitative RT-PCR reference genes is needed to accurately measure fluctuations in the expression of genes. Here, we evaluated candidate reference genes applicable for quantitative RT-PCR analysis of gene expression during proliferation and adipogenesis of human ASCs with the immunophenotype DLK1^+^/CD34^+^/CD90^+^/CD105^+^/CD45^−^/CD31^−^.

**Methods:**

We evaluated the applicability of 10 candidate reference genes (*GAPDH*, *TBP*, *RPS18*, *EF1A*, *TFRC*, *GUSB*, *PSMD5*, *CCNA2*, *LMNA* and *MRPL19*) using NormFinder, geNorm and BestKeeper software.

**Results:**

The results indicate that *EF1A* and *MRPL19* are the most reliable reference genes for quantitative RT-PCR analysis of proliferating ASCs. *PSMD5* serves as the most reliable endogenous control in adipogenesis. *CCNA2* and *LMNA* were among the least consistent genes.

**Conclusions:**

Applying these findings for future gene expression analyses will help elucidate ASC biology.

**Electronic supplementary material:**

The online version of this article (10.1186/s11658-019-0140-6) contains supplementary material, which is available to authorized users.

## Background

Adipose stromal cells (ASCs) are a major reservoir of adipocyte precursors in adipose tissues [1, 2]. Their heterogeneous population contains stem and progenitor cells, which are essential for adipose tissue development, regeneration and homeostasis [3]. Thus, ASCs are crucial for the maintenance of adipose tissue functions. They are also a viable source of material for cell-based therapies in regenerative medicine [4].

The large population of human ASCs with the immunophenotype DLK1^+^/CD34^+^/CD90^+^/CD105^+^/CD45^−^/CD31^−^ consists of adipose progenitor cells with high proliferative and adipogenic potential [5, 6]. Proliferation is a highly dynamic process involving many changes in gene expression occurring in response to extracellular signals and as a function of cell cycle progression regulated by specific cyclin-dependent kinase activities [7]. The differentiation of ASCs into adipocytes, also referred to as adipogenesis, gives rise to a new cell type [8]. A cascade of transcriptional regulators orchestrates adipogenesis. The expression of the adipogenic transcription factor peroxisome proliferator-activated receptor γ2 (PPAR γ2), which is both required and sufficient to drive adipogenesis, is a key event in the induction of the adipogenic differentiation program [9, 10]. The function of PPAR γ2 is tightly linked to members of the CCAAT/enhancer-binding protein (C/EBP) family of transcription factors [11]. The functional interaction of these factors induces changes in the expression of a large number of genes, leading to distinct morphological and biochemical alterations and eventually to the generation of adipocytes [12]. To accurately analyze the changes in gene expression during both proliferation and adipogenic differentiation of ASCs, appropriate endogenous controls are needed.

Valid reference genes are crucial for reliable gene expression analyses using quantitative RT-PCR [13, 14]. They serve as internal controls, allowing normalization, which counteracts inter-sample variations due to differences in RNA quantity and quality [13, 15]. State-of-the-art quantitative RT-PCR is essential for the validation of appropriate reference genes [16, 17].

A constitutive expression pattern qualifies a certain gene to act as internal control [15, 16, 18]. Commonly used reference genes include those coding for metabolic enzymes, such as glyceraldehyde-3-phosphate dehydrogenase (*GAPDH*), or components of the cytoskeleton, such as β-actin (*ACTB*) and β-2-microglobulin (*B2M*) [16, 17]. The 18S ribosomal RNA is also considered to be a reliable reference in quantitative RT-PCR experiments [19]. However, it is known that the expressions of genes coding for metabolic enzymes [20] and cytoskeleton components [21] are regulated and dependent on the physiological conditions or cellular state. In fact, no single gene exists which is constitutively expressed in all cell types under all experimental conditions [15, 18].

Thus, a careful validation of reference genes for given applications is mandatory. There is still a need for appropriate endogenous controls for quantitative RT-PCR analysis to cope with the strong fluctuations in gene expression during the complex processes of ASC proliferation and adipocyte differentiation. This study aimed to evaluate candidate reference genes reliable for quantitative RT-PCR analysis of proliferating and differentiating human ASCs to enable future elucidation of ASC biology.

## Methods

### Study design

We aimed to identify appropriate quantitative RT-PCR reference genes for the analysis of gene expression in the course of proliferation and adipogenesis of human ASCs.

### Isolation and cultivation of ASCs

Human ASCs were isolated from abdominal subcutaneous white adipose tissue (sWAT) samples obtained from four females undergoing routine elective plastic abdominal surgery at the Institute for Plastic and Reconstructive Surgery of the Medical University of Innsbruck. All patients gave their informed written consent. The study protocol was approved by the Ethics Committee of the Medical University of Innsbruck and complies with the Helsinki Declaration. The characteristics of the donors are given in Additional file 1: Table S1. Isolation and cultivation of ASCs was performed as described previously [6]. ASCs were maintained in PM4 medium, which is DMEM/F-12 medium (1:1) with HEPES and l-glutamine (Gibco) containing 33 μM biotin, 17 μM pantothenate 20 μg/ml ciprofloxacin, 2.5% FCS, 10 ng/ml EGF, 1 ng/ml bFGF and 500 ng/ml insulin. The ASCs were split at 70% confluence. Passage 3 ASCs were used for the whole study.

### Adipogenic differentiation

Adipogenesis was induced as described previously [6]. ASCs were expanded until confluence in PM4 medium and serum-starved for another 48 h in serum-free ASC medium consisting of DMEM/F-12 medium (1:1) with HEPES and l-glutamine (Gibco) containing 33 μM biotin, 17 μM pantothenate and 20 μg/ml ciprofloxacin. Adipogenesis was induced by the addition of differentiation medium, which was ASC medium containing 0.2 μM insulin, 0.5 mM IBMX, 0.25 μM dexamethasone, 2.5% FCS and 10 μg/ml transferrin. After 3 days, the medium was changed, and the cells were cultivated until day 14 in differentiation medium without IBMX. Cell extracts for western blot and gene expression analysis were taken at defined time points before and after induction of adipogenic differentiation. Oil-Red-O staining was performed to visualize lipid droplets of differentiated adipocytes. Briefly, cells were fixed in a 4% paraformaldehyde–PBS mixture for 30 min, washed with PBS, stained with 0.5% Oil-Red-O in a 60:40 isopropanol:water mix for 1 h, and finally washed with H_2_O.

### Proliferation monitoring

For the proliferation assays, 6-well plates were seeded with 60,000 cells/well. The cells were cultivated in PM4 medium containing 2.5% FCS (low mitogenic medium) and PM4 medium containing 10% FCS (high mitogenic medium) in the same experiment. Cell numbers were counted at defined time points using a Neubauer Chamber.

### Flow cytometry analysis

Fluorescence-activated cell sorting (FACS) was used to characterize the immunophenotype of human ASCs. 1 × 10^5^ cells per sample were simultaneously fixed and permeabilized using BD Cytofix/Cytoperm solution to prepare the cells for intracellular and surface staining. The cells were subjected to immunofluorescence staining using a panel of mouse monoclonal antibodies (BD Pharmingen): CD34-PE-Cy7 (#560710), CD105-PerCP-Cy5.5 (#560819), CD90-PE (#561970), CD45RA-FITC (#556626) and CD31-FITC (#555445). For DLK1 a primary rat monoclonal anti-human DLK1/PREF1 antibody (Adipogen, AG-25A-0091) along with the anti-rat-APC antibody (BD Pharmingen, #551019) was used. The labeled cells were measured using a FACS Canto II (BD Biosciences) and the data were analyzed using Flowing Software (http://www.flowingsoftware.com).

### RNA isolation and quantitative RT-PCR

Isolation of RNA was done using the RNeasy Plus Micro Kit (Qiagen, #74034) according to the manufacturer’s instructions. Briefly, cells were washed with PBS and lysed immediately with 350 μl RLT buffer per well. Genomic DNA (gDNA) was removed from the cell lysate by filtration through a gDNA Eliminator spin column (Qiagen). Subsequently, one volume 70% ethanol was added and the sample transferred onto the RNeasy MinElute binding column. Bound RNA was washed with RW1 buffer, RPE buffer and 80% ethanol. After elution, the yield and purity of the isolated RNA was determined spectrophotometrically. RNA integrity was confirmed using agarose gel electrophoresis.

For reverse transcription of RNA (1.5 μg per sample), we used the First Strand cDNA Synthesis Kit (Thermo Scientific, #K1622) according to the manufacturer’s guidelines. Subsequently, cDNA was diluted 1:15 and an aliquot of 5 μl was used for quantitative RT-PCR analysis.

Gene expression was measured with a LightCycler 480 (Roche) instrument using SYBR green chemistry and confirmed using corresponding non-template controls. Genomic DNA contamination was excluded using appropriate mock reverse-transcriptase controls. Primer were designed using QuantPrime [22] (http://quantprime.mpimp-golm.mpg.de/) and are given in Additional file 1: Table S3. Primer specificity was verified via melting curve analysis. The number of biological replicates (i.e., donors) per group was *n* = 3 (proliferation) and *n* = 4 (adipogenesis). All samples were measured in duplicate.

### Western blot analysis

ASCs were harvested in SDS lysis buffer and sonicated as described previously [6]. The protein concentration of the samples was determined with the Combat-Able Protein Assay Preparation Reagent Set (Thermo Scientific, #23215) and the Pierce BCA Protein Assay Kit (Thermo Scientific, #23227). Samples (10 μg total protein) were separated on 10% SDS-polyacrylamide gel, blotted onto a PVDF membrane and probed with polyclonal anti-perilipin antibodies (Cell Signaling Technology, #9349). To ensure equal loading and blotting, membranes were probed with an anti-β-actin antibody (Sigma Aldrich, AC15). Goat anti-rabbit IgG-HRP (DAKO) and anti-mouse IgG-HRP (Promega) served as secondary antibodies. Signals were detected using a chemiluminescence detection system.

### Software packages for quantitative RT-PCR analysis

Quantitative RT-PCR results were analyzed using three software packages, NormFinder, geNorm and BestKeeper, according to the developer’s instructions. The NormFinder algorithm was described by Andersen et al. [18] (software download: https://moma.dk/normfinder-software). The principles of the geNorm program were described by Vandesompele et al. [23] (software download: https://genorm.cmgg.be/). The BestKeeper software was described by Pfaffl et al. [24] (software download: http://www.gene-quantification.de/bestkeeper.html). Candidate reference genes were ranked based on the relevant algorithm.

### Statistics

Statistical analysis was performed using GraphPad Prism 5 (GraphPad) software. The number of different donors per group was *n* = 3 (proliferation) and *n* = 4 (adipogenesis). Comparison of two groups was achieved using Student’s *t*-test. *p* ≤ 0.05 was considered statistically significant.

## Results

### Proliferation and adipogenic differentiation of human ASCs

**Fig. 1:**
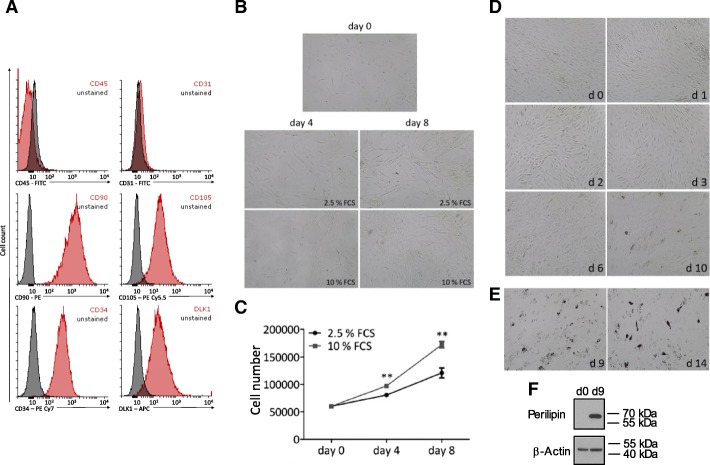
Characterization, proliferation and differentiation of ASCs. **a** – Characterization of ASCs via FACS analysis. 100,000 cells were fixed, permeabilized and analyzed for the expressions of the marker proteins CD45, CD31, CD90, CD105, CD34 and DLK1. Histograms of passage 3 ASCs are shown. Black histograms: Unstained control. Red histograms: Cells with specific antibody staining. Histograms are representative of 3 independent flow cytometry analyses using ASCs from different donors. **b** and **c** – Microphotographs (**b**) and growth curves (**c**) of proliferating ASCs cultivated in PM4 medium containing 2.5% FCS or 10% FCS. Each data point represents the average cell number of 3 different wells. Values are presented as means +/− SEM. **p < 0.01. **d** – Adipogenic differentiation of ASCs. Adipogenesis was induced on day 0 (d 0) and the morphology of the cells was documented using bright-field microscopy on the indicated days. **e** – The formation of lipid droplets was monitored using Oil-Red-O staining on days 9 and 14 post-induction of adipogenesis. **f** – The perilipin protein level was monitored via western blot analysis in undifferentiated (d 0) and differentiated (d 9) ASCs. Representative results from three independent experiments performed in ASCs derived from three different donors are shown

We evaluated candidate reference genes for quantitative RT-PCR-based gene expression studies of proliferating and differentiating human ASCs. First, we isolated ASCs from fresh abdominal sWAT samples obtained by incision from four female donors undergoing elective plastic abdominal surgery (Additional file 1: Table S1). The cells were stored in liquid nitrogen. To determine the purity of the ASC population, the cells were thawed and grown to passage 3. Afterwards the cells were permeabilized and subjected to multi-parameter detection FACS analysis using antibodies against established ASC marker proteins [6, 25]. The vast majority of the cells showed the characteristic ASC immunophenotype, DLK1^+^/CD34^+^/CD90^+^/CD105^+^/CD45^−^/CD31^−^ (Fig. 1a), which is expected for permeabilized passage 3 ASCs [6, 25].

The ASCs were then cultured in a low mitogenic medium (PM4 medium containing 2.5% FCS) and in a high mitogenic medium (PM4 medium containing 10% FCS). Proliferation was monitored by counting the ASC numbers on the indicated days (Fig. 1b and c). As expected, ASCs grown in high mitogenic medium showed a significantly higher proliferation rate compared to cells grown in low mitogenic medium.

For adipogenic differentiation, ASCs were grown to density arrest and starved in serum-free medium. Induction of adipogenesis by a hormone cocktail led to the characteristic morphological transformation of ASCs from a fibroblast-like morphology to rounded cells in the course of the first 72 h after induction (Fig. 1d). This is a hallmark of adipogenesis [26]. Differentiation was confirmed by detection of intracellular fat droplets (Fig. 1e) and the adipocyte-specific protein perilipin (Fig. 1f). The full western blot analysis is shown in Additional file 2: Figure S1.

### Selection and expression levels of reference genes

For quantitative RT-PCR analysis, total RNA was isolated from proliferating ASCs (3 donors) and from ASCs 0, 1, 2, 3, 6 and 10 days after induction of adipogenesis (4 donors). The yield ranged from 2 to 10 μg with a mean purity ratio (A260/A280) of 2.0.

We selected several candidate reference genes (Additional file 1: Table S2) to find the most reliable ones for RNA expression analysis in proliferating and differentiating ASCs. Standard curves for reference genes were processed based on proliferating ASCs whereas standards for adipogenic marker genes were performed on ASCs three days after the induction of adipogenesis (Additional file 3: Figure S2 and Additional file 4: Figure S3).

**Fig. 2:**
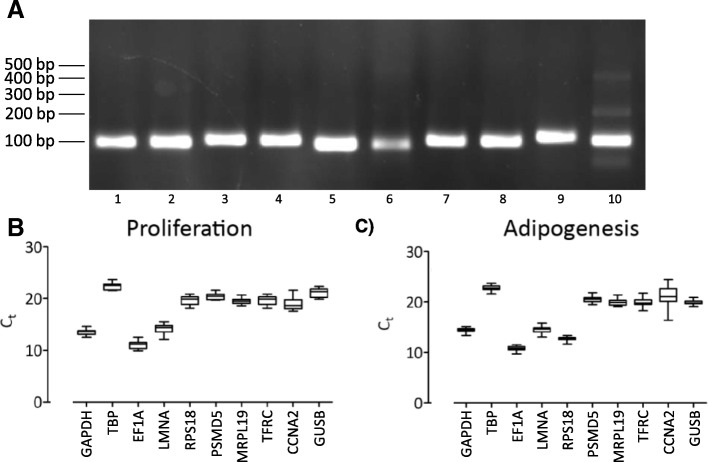
Primer specificity and average raw cycle thresholds. **a** – The amplification specificity of all candidate reference gene primer sets. cDNA was isolated from undifferentiated human ASCs. Lanes 1–10: GAPDH, TBP, EF1A, LMNA, RPS18, PSMD5, MRPL19, TCRF, CCNA2 and GUSB. B and C – Raw quantitative PCR C_T_ values for candidate reference genes during proliferation (**b**) and adipogenesis (**c**). Each gene was amplified in 15 (proliferation) or 24 (adipogenesis) different biological samples in duplicates. Values are presented as means +/− SEM

Quantitative RT-PCR for standards was performed using the classical 10-fold serial dilution method. The efficiencies (E) of reference and target gene primer sets had mean values of 101.9 +/− 2.81% and 103.9 +/− 3.80%, respectively (Additional file 4: Table S3). To confirm a specific PCR amplification, gel electrophoresis was performed. It showed only one PCR product at the predicted size (Fig. 2a). Additionally, melting curve analysis exhibited one clear peak for each primer pair (data not shown).

The tested reference genes showed various expression levels (Fig. 2b and c). To evaluate if the increased SDs within these four groups refer to significant outliers we performed Grubbs’ test, which detects outliers in a given data set and defines their significance. No significant outlier was detected with the threshold at *p* ≤ 0.05. Therefore, all samples were included for further analysis.

### Evaluation of appropriate reference genes for ASC proliferation and differentiation

**Fig. 3:**
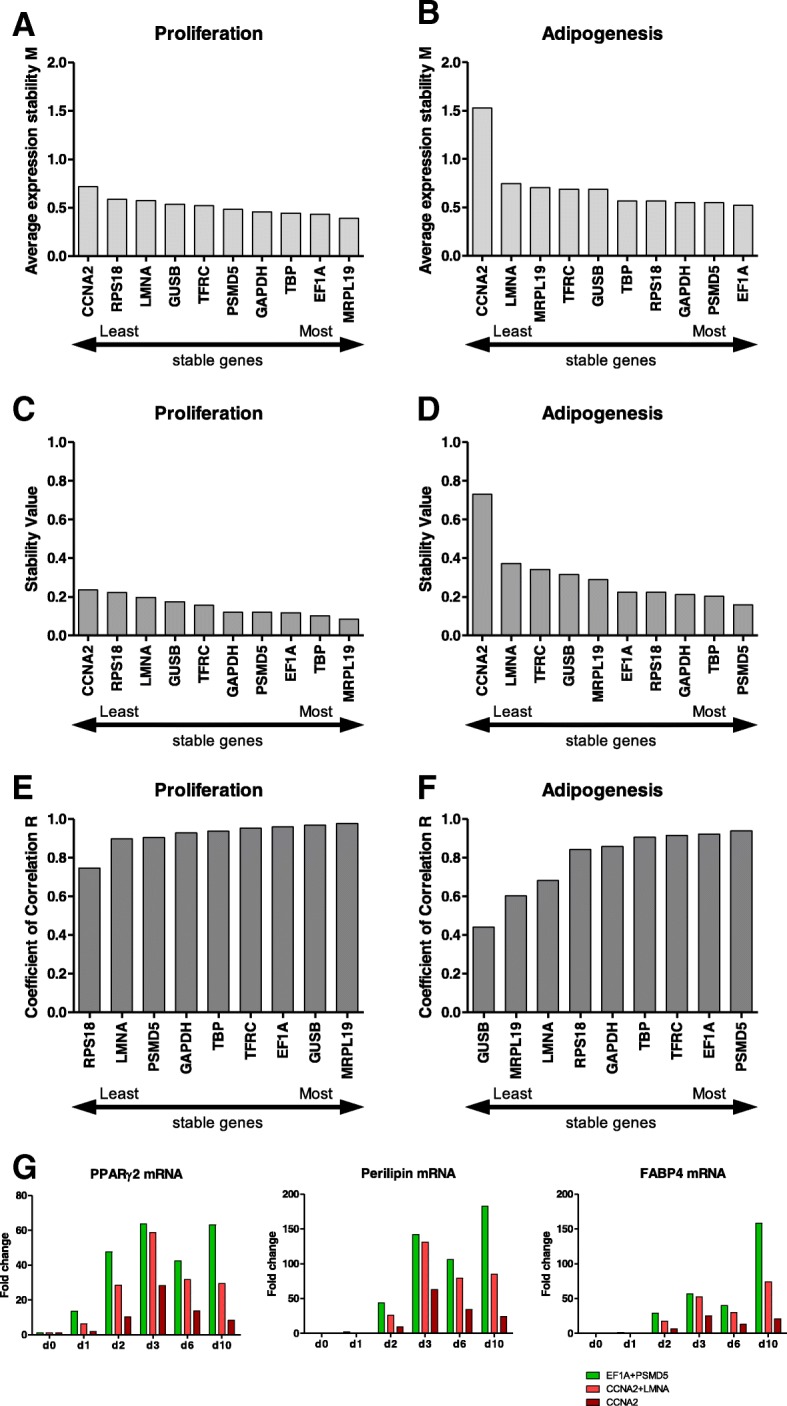
Analysis and ranking of candidate gene expression to determine the most stable reference genes in proliferation and adipogenesis. **a** and **b** – GeNorm analysis showing the stability value M of candidate reference genes in proliferating (**a**) and differentiating (**b**) ASCs. Lower values indicate more stable genes, higher values indicate less stable genes. **c** and **d** – NormFinder analysis showing the most stable reference genes in proliferating (**c**) and differentiating (**d**) ASCs. Lower values indicate more stable genes, higher values indicate less stable genes. **e** and **f** – BestKeeper analysis showing the most stable reference genes (based on their Pearson correlation coefficient) for proliferation (**e**) and differentiation (**f**). Higher values indicate more stable genes, lower values indicate less stable genes. *p* < 0.001 (exceptions: proliferation: RPS18 *p* = 0.002; adipogenesis: GUSB *p* = 0.03, MRPL19 *p* = 0.003). **g** – Effects of appropriate (green) and inappropriate (red) reference genes on the relative expressions of adipogenic marker genes in the course of adipogenesis are shown

The candidate reference genes selected in this study encode proteins in different functional classes so the chance that genes might be co-regulated is low [18]. To select optimal reference genes for ASC proliferation and differentiation, three different mathematical approaches (GeNorm, NormFinder and BestKeeper) were used:

GeNorm analysis ranks candidate reference genes with their lowest expression stability value (M-value) up to a threshold of 0.5. Genes with values exceeding 0.5 are considered unstable [27], although in heterogeneous cell populations, an M-value of 1.0 can also be accepted [27]. ASCs undergoing the differentiation process cannot be regarded as a homogeneous cell population compared to cycling ASCs. Therefore, the threshold was set to 0.5 for proliferating cells and 1.0 for differentiating cells. The M-values generated using the GeNorm software are presented in Fig. 3a and b.

The NormFinder algorithm calculates the stability value of each gene. Based on this analysis, the use of two candidate genes with the lowest stability is recommended (threshold 0.15) [18]. As shown in Fig. 3c, candidate genes (*MRPL19*, *TBP*, *EF1A*, *PMSD5* and *GAPDH*) meet the criteria defined by the normalization factor cut off value 0.15 for proliferating ASCs. NormFinder analysis revealed *MRPL19* and *TBP* to be the best combination of reference genes for proliferating ASCs (stability value 0.075). However, the stability values of candidate genes tested in differentiating ASCs failed to remain below the threshold of 0.15 (Fig. 3d). As mentioned above, these higher values might be due to the heterogeneity of differentiating cells. However, the combination of *PSMD5* and *TBP* changed the stability value to an acceptable number of 0.122.

BestKeeper analysis step-wisely excludes unsuitable candidate reference genes. After descriptive statistical analysis for each reference gene, candidates with a standard deviation above 1.0 are immediately excluded. Subsequently, pair-wise correlation analysis is performed to calculate the Pearson correlation co-efficient R for every reference gene. High R values are considered to indicate a stable gene expression pattern [24]. Analysis of C_T_ values of all candidate genes in proliferating ASCs revealed a SD (standard deviation) below 1.0 (data not shown). *CCNA2* was excluded from further calculations due to its high SD (0.89). Further analysis showed a strong correlation for all candidate genes (0.977 < R < 0.741; Fig. 3e). When we repeated BestKeeper analysis with the three most appropriate genes, *MRPL19*, *GUSB* and *EF1A*, the correlation even increased (0.985 < R < 0.987, Additional file 1: Table S4). After excluding *CCNA2* (SD = 1.5), candidate reference genes in adipogenic ASCs showed a rather weak correlation (0.920 < R < 0.437, Fig. 3f). However, examination of the three best candidates (*PSMD5*, *EF1A* and *TFRC*) revealed a strong correlation between these genes (0.969 < R < 0.935, Additional file 1: Table S4).

The impact of different reference genes on the expressions of genes of interest (GOIs) was evaluated in differentiating ASCs. Representative time-course experiments using the combinations *EF1A* + *PSMD5*, *CCNA2* + *LMNA* and only *CCNA2* as the reference gene(s) are shown in Fig. 3g. It is clear that the selection of the reference gene(s) has considerable influence on the measurement of GOI expression.

## Discussion

Cell cycle progression and differentiation of ASCs into mature adipocytes are highly orchestrated and associated with major changes in the gene expression pattern [7, 8, 11]. To measure transcriptional changes during these processes, reliable approaches are required [28].

Quantitative RT-PCR is an established and highly sensitive technique to measure the expression of a gene of interest [29]. Absolute and relative quantitation of gene expression are possible with this technique. The first approach requires a costly standard curve to determine the number of transcripts present in the sample, while the latter depends on appropriate reference genes for relative quantitation of gene expression [16, 17]. In this study, we combined the NormFinder, GeNorm and BestKeeper software packages [18, 23, 24] to define new reference genes for the comparison of gene expression in proliferating and adipogenic differentiating ASCs. Our results demonstrate the feasibility of this combinatorial approach. Similar results were obtained with all three programs.

*EF1A* and *MRPL19* were identified as the most stably expressed genes in the proliferating ASCs. The three algorithms identified *PSMD5* as the most stably expressed gene in adipogenically differentiating ASCs. *EF1A* was ranked by GeNorm and BestKeeper to be among the most stable reference genes for adipogenesis (Table 1). Commonly used endogenous control genes such as *GAPDH* showed an intermediate stability valuated during proliferation and adipogenic differentiation.

**Table 1:** Ranking of reference genes

	Proliferation			Adipogenesis		
Rank	GeNorm	NormFinder	BestKeeper	GeNorm	NormFinder	BestKeeper
1 (most stable)	MRPL19	MRPL19	MRPL19	EF1A	PSMD5	PSMD5
2	EF1A	TBP	GUSB	PSMD5	TBP	EF1A
3	TBP	EF1A	EF1A	GAPDH	GAPDH	TFRC
4	GAPDH	PSMD5	TCRF	RPS18	RPS18	TBP
5	PSMD5	GAPDH	TBP	TBP	EF1A	GAPDH
6	TFRC	TFRC	GAPDH	GUSB	MRPL19	RPS18
7	GUSB	GUSB	PSMD5	TFRC	GUSB	LMNA
8	LMNA	LMNA	LMNA	MRPL19	TFRC	MRPL19
9	RPS18	RPS18	RPS18	LMNA	LMNA	GUSB
10 (least stable)	CCNA2	CCNA2		CCNA2	CCNA2	

Our results underscore the context-dependent expression of reference genes and the requirement to find the most appropriate one(s) for given experimental conditions. Our findings are in agreement with those of other studies that identified *EF1A* and *TBP* as useful endogenous controls to analyze gene expression in differentiating mesenchymal stem cells [13, 28]. The experimental strategy of our study is a straightforward way to identify novel reference genes for quantitative RT-PCR analysis of proliferating and differentiating ASCs. This strategy has also been applied to mesenchymal stem cells of human [13] and rat origin [30].

## Conclusions

Our study identified *EF1A*, *MRPL19* and *PSMD5* as new quantitative RT-PCR reference genes suitable for measuring changes in gene expression during proliferation and adipogenesis of human DLK1^+^/CD34^+^/CD90^+^/CD105^+^/CD45^−^/CD31^−^ ASCs. The data suggest that *EF1A* and *MRPL19* are the most reliable reference genes for quantitative RT-PCR analysis of proliferating ASCs with that immunophenotype and *PSMD5* serves as the best endogenous control for gene expression analysis in the course of adipogenic differentiation of these cells. Applying these findings in future experiments will help elucidate ASC biology.

## Additional files

Additional file 1: **Table S1.** Donor characteristics. All donors were healthy formerly obese Caucasian women, who underwent routine abdominoplasty. None of the women suffered from diabetes, liver, renal or other severe metabolic diseases. None of the women had cancer. Clinical and anthropometric parameters are indicated. **Table S2.** Selected candidate reference genes. **Table S3.** Primer sequences. **Table S4.** Results of BestKeeper analysis. (DOCX 30 kb)

Additional file 2: **Figure S1.** Detection of perilipin via western blot analysis. **A –** SDS-PAGE was carried out as described in the Methods section to separate proteins in cellular lysates by molecular weight. A pre-stained molecular weight marker (M) was included (Thermo Scientific, #26616). After gel electrophoreses, the proteins were electro-blotted onto one polyvinyl-difluoride (PVDF) membrane. The transfer of pre-stained marker bands indicated a successful western blot procedure. **B –** Immobilized PVDF membrane-bound proteins were probed using a specific antibody against perilipin (Cell Signaling Technology, #9349) followed by incubation with an appropriate horseradish peroxidase-conjugated (HRP-conjugated) secondary antibody (goat anti-rabbit IgG-HRP, DAKO). Signal development was achieved by applying the enhanced chemo-luminescence (ECL) substrate. The generated light signal was detected by exposure of the PVDF membrane to a X-ray film. Marker bands were manually transferred onto the X-ray film by adjusting the PVDF membrane and the X-ray film according to specific marks located in the film cassette. The corresponding X-ray film to the PVDF membrane shown in (A) is presented. **C –** To ensure equal loading, the same PVDF membrane was re-probed using a specific anti-β-actin antibody (Sigma Aldrich, AC15) followed by incubation with an HRP-conjugated secondary antibody (anti-mouse IgG-HRP). After applying the ECL substrate, the membrane was exposed to an X-ray film. The corresponding X-ray film to the PVDF membrane shown in (A) is presented. The molecular weight is given in kilo Daltons (kDa). (PDF 99 kb)

Additional file 3: **Figure S2.** Standard curves for reference genes. (PDF 446 kb)

Additional file 4: **Figure S3.** Standard curves for target genes. (PDF 81 kb)

## Abbreviations

ASCs: Adipose stromal cells
C/EBP: CCAAT/enhancer-binding protein
CCNA2: Cyclin A2
DLK-1: Delta-like protein 1
EF1A: Elongation factor alpha
EGF: Epidermal growth factor
FCS: Fetal Calf Serum
FGF: Fibroblast growth factor
GAPDH: Glyceraldehyde-3-phosphate dehydrogenase
gDNA: Genomic DNA
GOI: Gene of interest
GUSB: Glucuronidase beta
LMNA: Lamin A
MRPL19: Mitochondrial ribosomal protein 19
PPARγ2: Peroxisome proliferator-activated receptor γ2
PSMD5: Proteasome 26S subunit, non-ATPase 5
RPS18: Ribosomal protein S18
RT-PCR: Reverse transcription polymerase chain reaction
SD: Standard deviation
SEM: Standard error of the mean
SVF: Stromal vascular fraction
sWAT: Subcutaneous white adipose tissue
TBP: TATA-box binding protein
TFRC: Transferrin receptor
