# Supplementary material for: Reliable reference genes for expression analysis of proliferating and adipogenically differentiating human adipose stromal cells
Source: Cell Mol Biol Lett. 2019 Feb 15;24:14. doi: 10.1186/s11658-019-0140-6 (PMC6377720; doi:10.1186/s11658-019-0140-6)
Supplement: Supplementary file 1 — Table S1. Donor characteristics. All donors were healthy formerly obese Caucasian women, who underwent routine abdominoplasty. None of the women suffered from diabetes, liver, renal or other severe metabolic diseases. None of the women had cancer. Clinical and anthropometric parameters are indicated. Table S2. Selected candidate reference genes. Table S3. Primer sequences. Table S4. Results of BestKeeper analysis. (DOCX 30 kb) [file 11658_2019_140_MOESM1_ESM.docx]

**Supplementary Material**

Supplementary Table S1:

| Female donor | Age [years] | Body mass index [kg/m^2^] |
| --- | --- | --- |
| #1 | 35 | 21,89 |
| #2 | 63 | 24,13 |
| #3 | 32 | 24,45 |
| #4 | 62 | 24,35 |

Donor characteristics. All donors were healthy formerly obese Caucasian women, who underwent routine abdominoplasty. None of the women suffered from diabetes, liver, renal or other severe metabolic diseases. None of the women had cancer. Clinical and anthropometric parameters are indicated.

Supplementary Table S2:

| Gene symbol | Gene name | Accession number | Function | Ref. |
| --- | --- | --- | --- | --- |
| *GAPDH* | Glyceraldehyde-3-phosphate dehydrogenase | NM_002046.3 | Involved in glycolysis | [[1](#_ENREF_1)] |
| *TBP* | TATA-box binding protein | NM_001172085.1 | Transcription factor | [[2](#_ENREF_2)] |
| *EF1A* | Elongation factor alpha | NM_001402.5 | Translation elongation factor | [[2](#_ENREF_2)] |
| *TFRC* | Transferrin receptor | NM_003234.2 | Cellular uptake of iron | [[2](#_ENREF_2)] |
| *GUSB* | Glucuronidase beta | NM_000181.3 | Galactosidase | [[3](#_ENREF_3)] |
| *PSMD5* | Proteasome 26S subunit, non-ATPase 5 | NM_005047.2 | Chaperone protein involved in 26S proteasome assembly | [[4](#_ENREF_4)] |
| *RPS18* | Ribosomal protein S18 | NM_022551.2 | Ribosomal subunit | [[5](#_ENREF_5)] |
| *CCNA2* | Cyclin A2 | NM_001237.3 | Cell cycle control | [[6](#_ENREF_6)] |
| LMNA | Lamin A | NM_170707.2 | Mediates nuclear stability, chromatin structure and gene expression | [[7](#_ENREF_7)] |
| *MRPL19* | Mitochondrial ribosomal protein 19 | NM_014763.3 | Protein synthesis | [[3](#_ENREF_3)] |

Selected candidate reference genes.

Supplementary Table S3:

| Gene | Sequence | Exon-exon boarder | Amplicon  [bp] | Efficiency  [%] |
| --- | --- | --- | --- | --- |
| C/EBPA_f | GACAGTGGGAGAGACGGATAAG | yes | 122 | 110.48 |
| C/EBPA_r | TGATTCCCATTTGTGCCTCGTG |  |  |  |
| C/EBPB_f | CTCTGTGTGGTGCTAACTTGCC | yes | 98 | 99.54 |
| C/EBPB_r | TGTGCCTGAGACATGAATGAGC |  |  |  |
| C/EBPD_f | TCAGCAACGACCCATACCTCAG | no | 114 | 80.55 |
| C/EBPD_r | CTTTGCGCTCCTATGTCCCAAG |  |  |  |
| CDK4_f | AAGGTAACCCTGGTGTTTGAGC | yes | 111 | 117.18 |
| CDK4_r | AAACTGGCGCATCAGATCCTTG |  |  |  |
| CCNA2_f | TTGATAGATGCTGACCCATACCTC | yes | 106 | 95.53 |
| CCNA2_r | ATGATTCAGGCCAGCTTTGTCC |  |  |  |
| Cyclin D1_f | CCCTCGGTGTCCTACTTCAAATG | yes | 98 | 106.61 |
| Cyclin D1_r | TTCTGTTCCTCGCAGACCTC |  |  |  |
| EF1alpha_f | CTACCACCAACTCGTCCAACTG | yes | 88 | 103.05 |
| EF1alpha_r | CAACAGGAACAGTACCAATACCAC |  |  |  |
| FABP4_f | CCTGGTACATGTGCAGAAATGGG | yes | 94 | 104.48 |
| FABP4_r | GCCTTTCATGACGCATTCCAC |  |  |  |
| GAPDH_f | TCCTGCACCACCAACTGCTTAG | yes | 86 | 99.96 |
| GAPDH_r | TGGACTGTGGTCATGAGTCCTTC |  |  |  |
| GUSB_f | AGAAGTGGTGCGTAGGGACAAG | yes | 120 | 96.53 |
| GUSB_r | TTGGTGTGAGCGATCACCATCTTC |  |  |  |
| LMNA_f | GCAAGACCCTTGACTCAGTAGC | yes | 111 | 93.95 |
| LMNA_r | CACCCTCCTTCTTGGTATTGCG |  |  |  |
| MRPL19_f | GGAATGTTATCGAAGGACAAGGTG | yes | 107 | 99.13 |
| MRPL19_r | CAAGCTATCATCCAGCCGTTTC |  |  |  |
| Perilipin_f | GACAACGTGGTGGACACAGT | yes | 97 | 107.66 |
| Perilipin_r | CTGGTGGGTTGTCGATGTC |  |  |  |
| PPARG_f | CTGTCATTATTCTCAGTGGAGACC | yes | 82 | 104.71 |
| PPARG_r | GCTTGTAGCAGGTTGTCTTGAATG |  |  |  |
| PPARg2_f | ATG GGT GAA ACT CTG GGA GA | yes | 246 | Not determined |
| PPARg2_r | TGG AAT GTC TTC GTA ATG TGG A |  |  |  |
| PSMD5_f | GGTGTAGCTGTAGACACAGTTGG | yes | 89 | 102.26 |
| PSMD5_r | AAGCGTTCAAAGCGAGTTCCTG |  |  |  |
| RPS18_f | GGCCGAAGATATGCTCATGTGG | yes | 100 | 99.42 |
| RPS18_r | TGGTGATCACACGTTCCACCTC |  |  |  |
| TBP_f | GGATAAGAGAGCCACGAACCAC | yes | 90 | 121.83 |
| TBP_r | CCAGTCTGGACTGTTCTTCACTC |  |  |  |
| TFRC_f | TTCCACCATCTCGGTCATCAGG | yes | 101 | 92.24 |
| TFRC_r | GGGACAGTCTCCTTCCATATTCCC |  |  |  |

Primer sequences.

Supplementary Table S4:

|  | Proliferation | | Adipogenesis | |
| --- | --- | --- | --- | --- |
| Rank | Gene | R | Gene | R |
| 1 | MRPL19 | 0.985 | PSMD5 | 0.935 |
| 2 | GUSB | 0.985 | EF1A | 0.969 |
| 3 | EF1A | 0.987 | TFRC | 0.969 |

Results of BestKeeper analysis.

**Supplementary References**

[1] M.L. Wong, J.F. Medrano, Real-time PCR for mRNA quantitation, Biotechniques 39 (2005) 75-85.

[2] E.d.L. Rebouças, J.J.d.N. Costa, M.J. Passos, J.R.d.S. Passos, R.v.d. Hurk, J.R.V. Silva, Real time PCR and importance of housekeepings genes for normalization and quantification of mRNA expression in different tissues, Brazilian Archives of Biology and Technology 56 (2013) 143-154.

[3] R.E. McNeill, N. Miller, M.J. Kerin, Evaluation and validation of candidate endogenous control genes for real-time quantitative PCR studies of breast cancer, BMC Mol Biol 8 (2007) 107.

[4] Q. Deveraux, C. Jensen, M. Rechsteiner, Molecular cloning and expression of a 26 S protease subunit enriched in dileucine repeats, J Biol Chem 270 (1995) 23726-23729.

[5] A.A. Ilin, A.A. Malygin, G.G. Karpova, Ribosomal protein S18e as a putative molecular staple for the 18S rRNA 3'-major domain core, Biochim Biophys Acta 1814 (2011) 505-512.

[6] T. Gao, Y. Han, L. Yu, S. Ao, Z. Li, J. Ji, CCNA2 is a prognostic biomarker for ER+ breast cancer and tamoxifen resistance, PLoS One 9 (2014) e91771.

[7] K. Mekhail, D. Moazed, The nuclear envelope in genome organization, expression and stability, Nat Rev Mol Cell Biol 11 (2010) 317-328.
