# Supplementary figures and images for: Reliable reference genes for expression analysis of proliferating and adipogenically differentiating human adipose stromal cells
Source: Cell Mol Biol Lett. 2019 Feb 15;24:14. doi: 10.1186/s11658-019-0140-6 (PMC6377720; doi:10.1186/s11658-019-0140-6)

**A)**

PVDF membrane

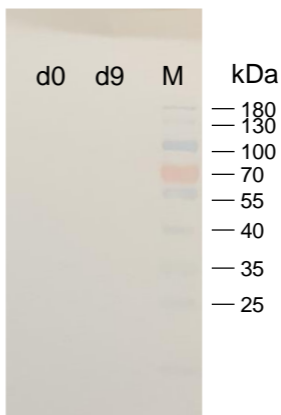

**B)**

Detection of Perilipin  
(X-ray film)

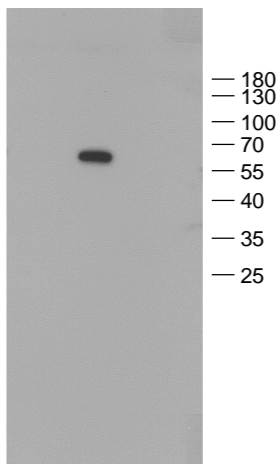

**C)**

Detection of  $\beta$ -Actin  
(X-ray film)

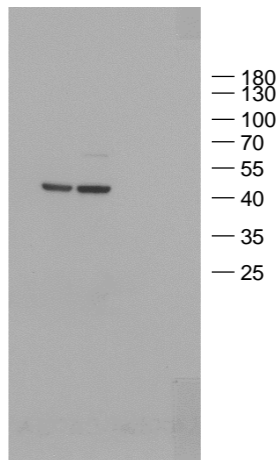

Supplement: Supplementary file 2 — Figure S1. Detection of perilipin via western blot analysis. A – SDS-PAGE was carried out as described in the Methods section to separate proteins in cellular lysates by molecular weight. A pre-stained molecular weight marker (M) was included (Thermo Scientific, #26616). After gel electrophoreses, the proteins were electro-blotted onto one polyvinyl-difluoride (PVDF) membrane. The transfer of pre-stained marker bands indicated a successful western blot procedure. B – Immobilized PVDF membrane-bound proteins were probed using a specific antibody against perilipin (Cell Signaling Technology, #9349) followed by incubation with an appropriate horseradish peroxidase-conjugated (HRP-conjugated) secondary antibody (goat anti-rabbit IgG-HRP, DAKO). Signal development was achieved by applying the enhanced chemo-luminescence (ECL) substrate. The generated light signal was detected by exposure of the PVDF membrane to a X-ray film. Marker bands were manually transferred onto the X-ray film by adjusting the PVDF membrane and the X-ray film according to specific marks located in the film cassette. The corresponding X-ray film to the PVDF membrane shown in (A) is presented. C – To ensure equal loading, the same PVDF membrane was re-probed using a specific anti-β-actin antibody (Sigma Aldrich, AC15) followed by incubation with an HRP-conjugated secondary antibody (anti-mouse IgG-HRP). After applying the ECL substrate, the membrane was exposed to an X-ray film. The corresponding X-ray film to the PVDF membrane shown in (A) is presented. The molecular weight is given in kilo Daltons (kDa). (PDF 99 kb) [file 11658_2019_140_MOESM2_ESM.pdf]

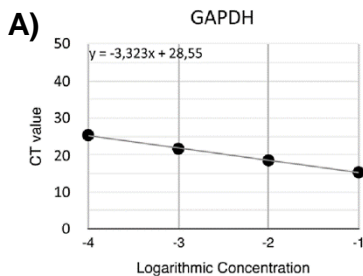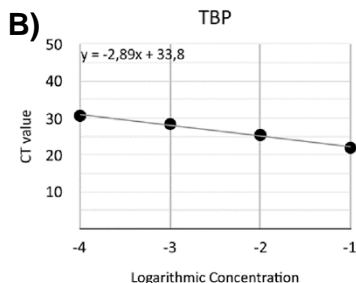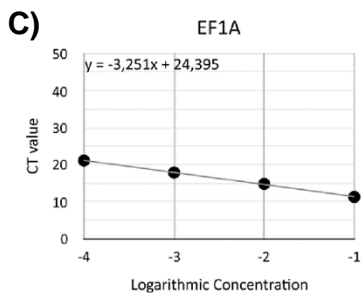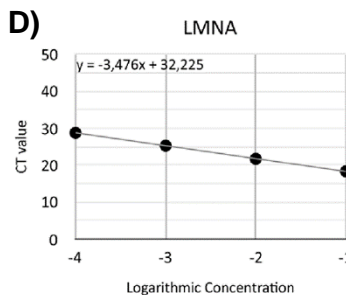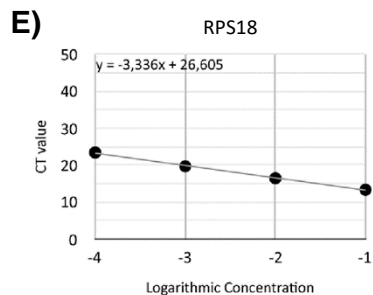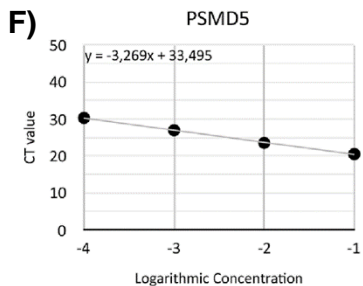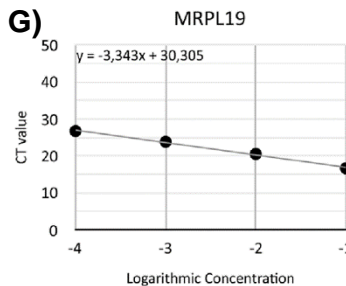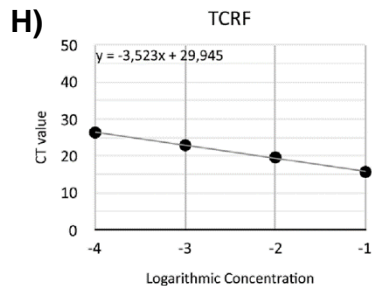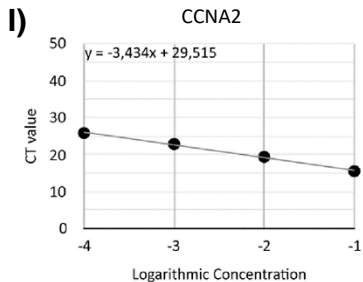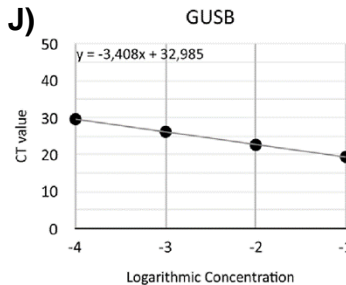

Supplement: Supplementary file 3 — Figure S2. Standard curves for reference genes. (PDF 446 kb) [file 11658_2019_140_MOESM3_ESM.pdf]

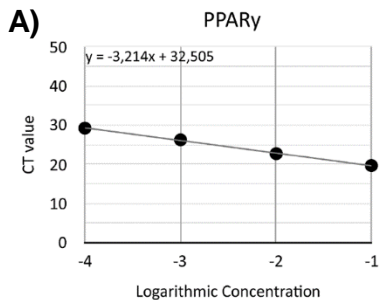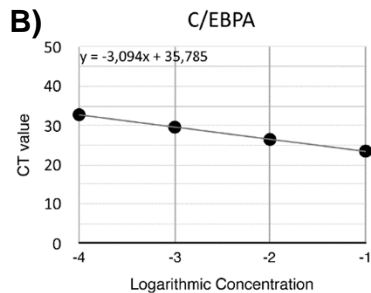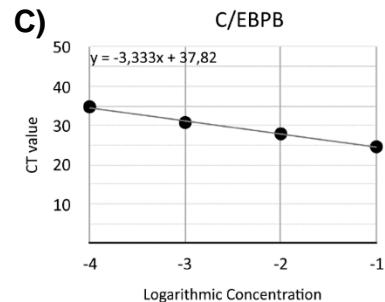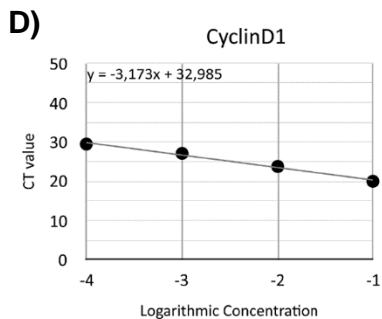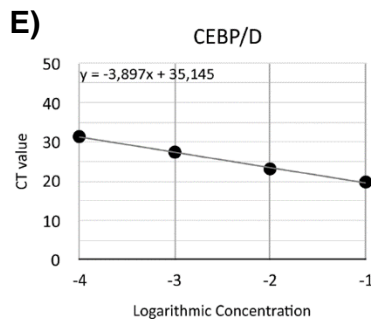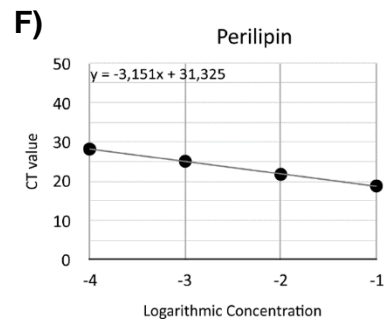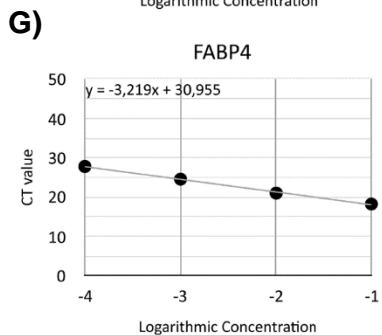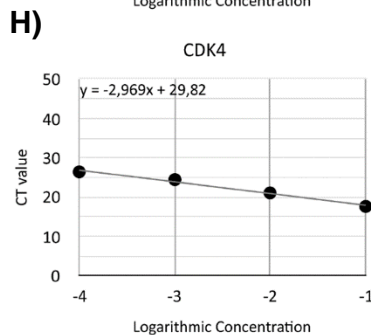

Supplement: Supplementary file 4 — Figure S3. Standard curves for target genes. (PDF 81 kb) [file 11658_2019_140_MOESM4_ESM.pdf]
